# Supplementary figures and images for: Mechanisms for Electron Uptake by Methanosarcina acetivorans during Direct Interspecies Electron Transfer
Source: mBio. 2021 Oct 5;12(5):e02344-21. doi: 10.1128/mBio.02344-21 (PMC8546582; doi:10.1128/mBio.02344-21)

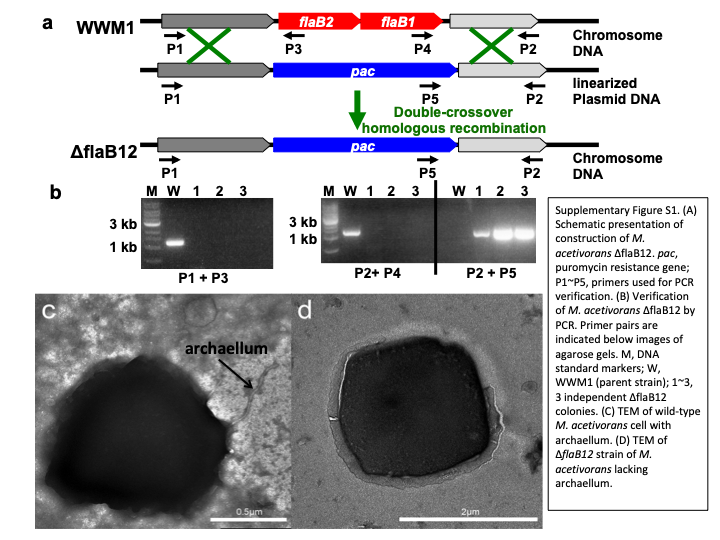

Supplement: FIG S1 [file mbio.02344-21-sf001.tif]
